# SiOx/C Composite Anode for Lithium-Ion Battery with Improved Performance Using Graphene Quantum Dots and Carbon Nanoparticles

**DOI:** 10.3390/molecules29112578

**Published:** 2024-05-30

**Authors:** Sung Won Hwang

**Affiliations:** Department of System Semiconductor Engineering, Sangmyung University, Cheonan 31066, Republic of Korea; sungwon@smu.ac.kr

**Keywords:** graphene quantum dot, composite anode, lithium-ion battery, carbon nanoparticle, SiOx

## Abstract

In this study, a composite was manufactured by mixing graphene quantum dots, silicon oxide, and carbon nanoparticles, and the characteristics of the anode materials for secondary batteries were examined. To improve the capacity of the graphene quantum dot (GQD) anode material, the added silicon oxide content was varied among 0, 5, 10, 15, and 30 wt%, and carbon nanoparticles were added as a structural stabilizer to alleviate silicon oxide volume expansion. The physical properties of the prepared GQD/SiOx/C composite were investigated through XRD, SEM, EDS, and powder resistance analysis. Additionally, the electrochemical properties of the manufactured composite were observed through an analysis of the charge–discharge cycle, rate, and impedance of a lithium secondary battery. In the GQD/SiOx/C composite, by adding carbon nanoparticles, an internal cavity was formed that can alleviate the volume expansion of silicon oxide, and the carbon nanoparticles and silicon oxide particles were uniformly distributed. The formed internal cavity had a silicon oxide content of 5 wt%. Low initial efficiency was observed, and above 30 wt%, low cycle stability was observed. The GQD/SiOx/C composite with 15 wt% of silicon oxide added showed an initial discharge capacity of 595 mAh/g, a capacity retention rate of 92%, and a rate characteristic of 81 at 2 C/0.1 C. Silicon oxide was added to improve the capacity of the anode material, and carbon nanoparticles were added as a structural stabilizer to buffer the volume change of the silicon oxide. To use GQD/SiOx/C composite as a highly efficient anode material, the optimal silicon oxide content and carbon nanoparticle mechanism as a structural stabilizer were discussed.

## 1. Introduction

The electric vehicle market is expected to grow more than 40 times by 2030. Existing lithium secondary batteries have a limited energy density of 810 Wh/L, but next-generation lithium secondary batteries require an energy density of over 1000 Wh/L, so the need to increase battery capacity is emerging. The growth of the electric vehicle market continues to increase the demand for lithium secondary batteries, and lithium secondary batteries are widely used due to their advantages, such as high energy density, long cycle life, and high stability [1,2,3]. However, existing lithium secondary batteries mainly use graphite anode materials with low capacity (374 mAh/g), making it difficult to meet high energy-density requirements [4,5,6]. To meet the market demand for secondary batteries with high energy density, research is being actively conducted on materials and structures that improve the energy density of lithium secondary batteries [7,8,9,10]. Silicon oxide (SiOx) is being widely studied as a negative electrode material to improve the energy density of lithium secondary batteries. Silicon oxide has a higher capacity than carbon materials and has better cycle stability and initial efficiency than silicon (Si) [11,12,13,14]. However, silicon oxide has low electrical conductivity when charging and discharging. Silicon oxide causes continuous formation and change of the SEI (solid electrolyte interphase) layer at the interface with the electrolyte due to volume expansion, resulting in low initial efficiency and rapid capacity reduction [15,16,17]. Silicon oxide reacts with the electrolyte during the initial lithiation process to form lithium oxide (LiO) and lithium silicate (LixSiOy), which buffers large volume changes and improves cycle performance. Nevertheless, the formed lithium oxide and lithium silicate consume lithium ions in an irreversible reaction and cause volume expansion of the silicon oxide. As a result, the structure becomes unstable and a non-uniform SEI layer is formed, which reduces electrochemical performance. To solve this problem, research has been conducted to construct a silicon oxide–carbon composite by mixing carbon materials with high electrical conductivity and a crystal structure [18,19,20,21]. The carbon in these composites acts as a conductive buffer and improves the electrical conductivity of silicon oxide. In addition, in the case of a carbon structure with a high crystal structure, the volume expansion of silicon oxide is suppressed, and cycle stability is improved. However, as the content of silicon oxide increases, the volume expansion strain also increases, and thus, the stability of the composite structure deteriorates. To solve this problem, a silicon oxide–carbon composite using a porous structure is employed to improve structural stability by minimizing mechanical stress due to the volume expansion of silicon oxide. This porous structure shares space with silicon oxide and buffers mechanical stress caused by volume changes during charging and discharging, improving structural stability. This porous structure provides a high contact area between the electrode and electrolyte because the composite has a high surface area due to micro-pores, thereby reducing the distance of lithium-ion movement and increasing the rate of lithium insertion and desorption. The porous structure can, thus, lead to improved reversible capacity, cycle performance, and speed. However, limitations have been found in the application of anode materials because complex manufacturing processes and structural control make it difficult to form porous structures. Therefore, to actually apply the silicon oxide–carbon composite, it is advantageous to manufacture it using a material with a stable structure. To form a porous structure, a composite was prepared using carbon nanoparticles with a large specific surface area and high electrical conductivity and graphene quantum dots as a structural stabilizer. Carbon nanoparticles are composed of 96–98% carbon in colloidal fine particles; they have a large surface area and highly conductive surface. Therefore, the composite has a porous structure due to the fine carbon nanoparticles, which improve the electrical performance by buffering the volume expansion of silicon oxide. In this study, a porous silicon oxide–carbon composite was prepared by mixing graphene quantum dots, silicon oxide, and carbon nanoparticles; the electrochemical properties of the prepared composite were analyzed according to the silicon oxide content. With the addition of silicon oxide, the initial efficiency and rate characteristics during the charging and discharging of the composite improved, and as the content of silicon oxide increased, the electric capacity improved. Through this, the effect of porous structure formation by carbon nanoparticles in the composite on silicon oxide was examined. In addition, the optimal silicon oxide content was derived through the electrochemical analysis of the composite; a correlation between electrochemical properties depending on content was determined.

## 2. Results and Discussion

### 2.1. Characterization of GQD/SiOx/C Composite

**Figure 1:**
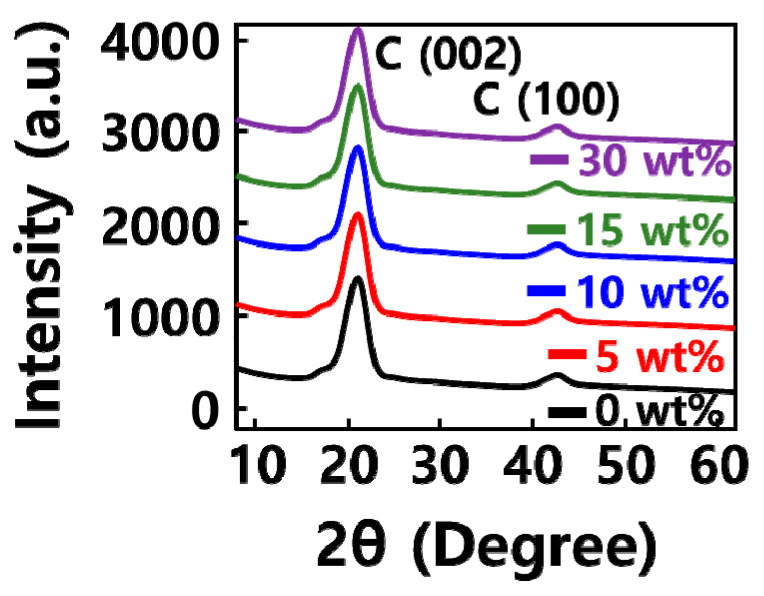
XRD pattern of GQD/SiOx/C composite-based anode.

Figure 1 shows the XRD pattern results of the GQD/SiOx/C composite. In the case of carbon particles, wide amorphous peaks appeared at 26° and 44°; the peaks represented crystal planes of C(002) and C(100). Silicon oxide showed broad, low peaks, characteristic of an amorphous structure, at 28.35, 47.24, and 56.23°. Because it forms an amorphous SiO_2_ structure in silicon oxide, Si showed main lattices of Si(111), Si(220), and Si(311). In the GQD/SiOx/C composite, peaks at 25.9 and 55.27° were observed regardless of the content, indicating that the crystal structure of C (002) and C (004) is the crystal structure of carbon [22]. Since the complex was formed using pitch, it was presumed that the carbon structure crystallized during the carbonization process. Carbon nanoparticles were manufactured during heat treatment through the pitch; the XRD peak that appears is a structural characteristic of carbon nanoparticles [23]. And since no peaks other than the carbon peak were observed, it was determined that silicon oxide and pitch reacted during the heat-treatment process, and no structural change to the SiC occurred. As a result of XRD analysis, it was confirmed that the carbon strength decreased as the silicon oxide content increased in the order of 0, 10, and 30 wt%. This is because, as the content of silicon oxide inside the composite increased, the content of carbon decreased [24].

**Figure 2:**
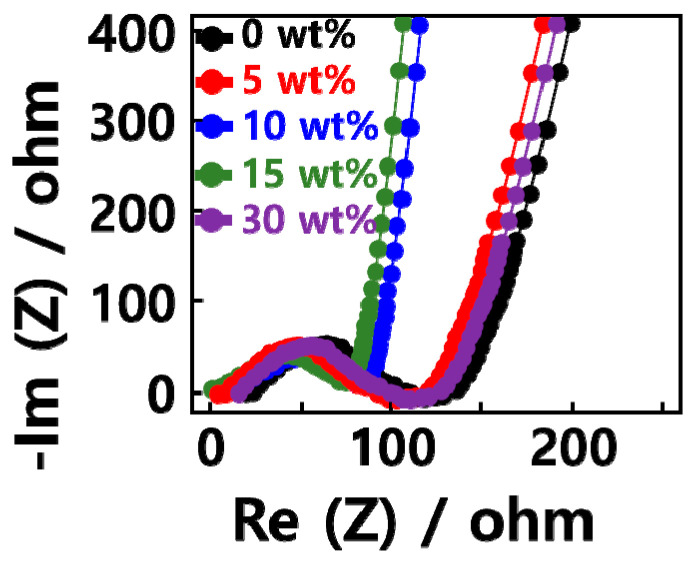
Nyquist—plots of the GQD/SiOx/C composite anode.

**Figure 3:**
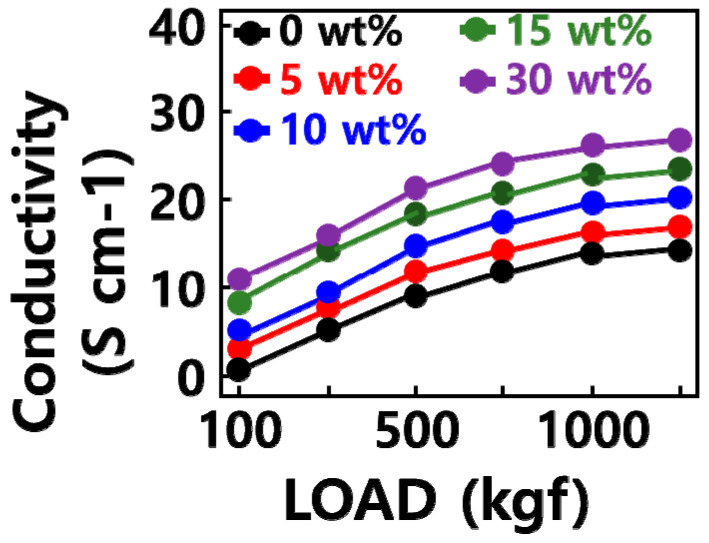
Electrical conductivity of the GQD/SiOx/C composite.

Figure 2 shows Nyquist plot characteristic results of a lithium secondary battery using GQD/SiOx/C composite as anode material. In the high-frequency region, ohmic resistance due to external connections, contact resistance, and ionic conduction in the electrolyte can be observed. In the low-frequency region, resistance due to charge movement can be observed. It was confirmed that the resistance decreased in the order of 30, 15, and 10 wt% for the complexes with high content. In the GQD/SiOx/C composite, the resistance value decreased through a smaller semicircle in the order of 15, 10, and 0 wt%. This is the opposite of the electrical conductivity of the composite shown in Figure 3, in which the content of silicon oxide increases. This shows that there is a difference between the electrical conductivity of the composite and the electronic resistance of the anode material. Inside the composite, cavities are formed due to the high specific surface area of the graphene quantum dots and carbon nanoparticles. As the silicon oxide content increased, the cavities inside the composite decreased, thereby improving electrical conductivity. However, when the content of silicon oxide increased during charging and discharging, the stability of the electrode decreased due to the volume expansion of the silicon oxide. So, the resistance increased, and the electrical conductivity decreased [25]. In addition, when comparing the degree of ion diffusion according to the content of silicon oxide in the GQD/SiOx/C composite, it was found that the slopes of the 0 and 15 wt% straight lines for the composite were high; this is advantageous for the diffusion of lithium ions. However, when the silicon oxide content of the GQD/SiOx/C composite was 30 wt%, a low straight-line slope was observed, which is believed to be due to the low ionic conductivity of the GQD/SiOx/C-20 composite. Low ionic conductivity is usually found in silicon-based anode materials; it reduces the structural stability of the GQD/SiOx/C-20 composite because the internal cavity of the composite cannot accommodate the volume change during charge and discharge of silicon oxide. Based on these results, the silicon oxide content of the GQD/SiOx/C composite was judged to be at an appropriate level of 15 wt%.

Figure 3 shows the electrical conductivity of the GQD/SiOx/C composite according to the SiOx content. The carbon nanoparticles used had a high electrical conductivity of 24 S/cm, while the silicon oxide was an insulator and had an electrical conductivity close to 0 S/cm. The carbon material showed an electrical conductivity of 1.6 S/cm, and the addition of carbon nanoparticles with high electrical conductivity increased the GQD/SiOx/C−0 composite approximately 8.3 times. Adding silicon oxide to the GQD/SiOx/C composite improved the electrical conductivity approximately 1.9 times, which is believed to be due to a decrease in the volume of the cavities inside the composite structure and an increase in composite density [26].

**Figure 4:**
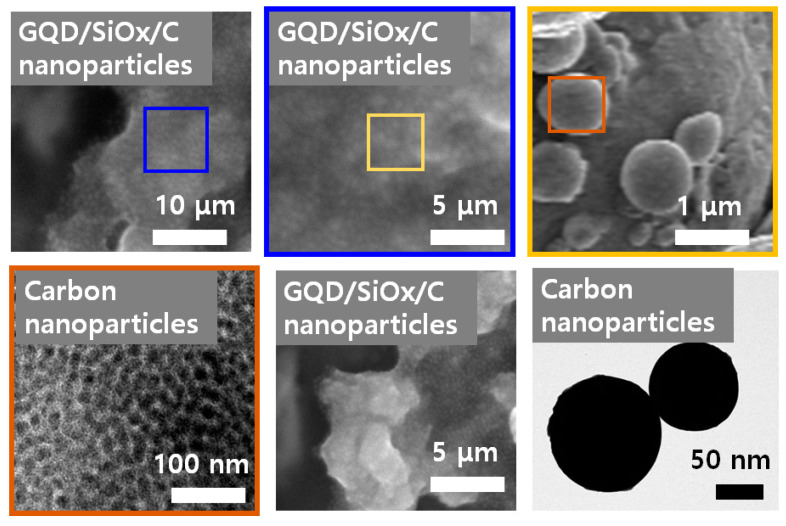
SEM and TEM images of the GQD/SiOx/C composite.

Figure 4 shows SEM results for the shape of the GQD/SiOx/C composite material. Silicon oxide is a SiO_2_ structure containing silicon particles; the small particles are clustered together and have a size of about 1 μm. In the SEM analysis image of the carbon nanoparticles, nanoparticles with uniform sizes of 50 to 100 nm can be observed; as shown in the high-magnification image, primary particles of 30 to 50 nm were aggregated. These FIB−SEM results confirm the internal structure of the GQD/SiOx/C composite, obtained through FIB milling, and the dispersion structure of silicon oxide as the silicon oxide content increases. Both GQD/SiOx/C composites have internal cavities that act as buffers against volume changes in silicon oxide during charging and discharging, preventing defects in the composite. Additionally, as the content of silicon oxide in the composite increased, the aggregate size and distribution of silicon oxide increased. As the SiOx content in the GQD/SiOx/C composite increased, the C content relatively decreased, and the Si content increased accordingly. The Mg was observed in the GQD/SiOx/C−20 composite because the utilized SiOx is alloyed with Mg. Therefore, it is believed that exposure to the surface took place during the FIB milling process.

### 2.2. Electrochemical Analysis of GQD/SiOx/C Composite Anode Material

**Figure 5:**
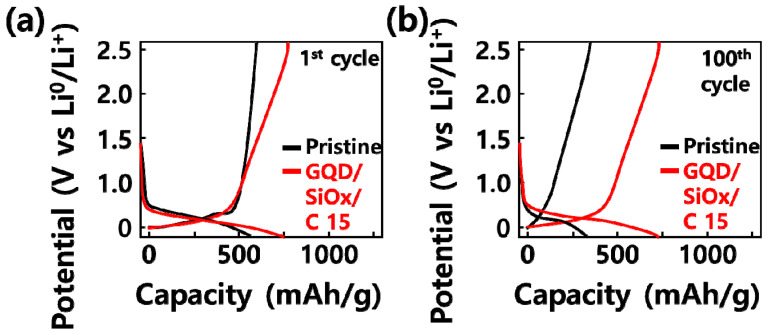
Comparison of electrochemical performances of pristine GQD/SiOx/C composites. The discharge-charge profiles at 100 mA·g^−1^ (**a**) 1st cycle and (**b**) 100th cycle.

Figure 5 shows the GQD/SiOx/C composite applied as an anode material for a lithium secondary battery and manufactured with different silicon oxide contents of 0, 5, 10, 15, and 30 wt%; corresponding charge and discharge characteristics are indicated. Initial charge–discharge results were tested between 0.01 V and 4 V depending on the silicon composite content. The SiOx-added composite shows a flat curve at 0.1 V, which is believed to be because a reaction between lithium ions and silicon occurred inside the composite [27]. The initial charge–discharge measurement results showed that the initial charge capacity improved as the SiOx content of the composite increased because the content of SiOx, which has high capacity, increased. Table 1 shows the GQD/SiOx/C composite synthesis conditions of the materials. Table 2 shows the initial cycle charge–discharge capacity at 0.1 C of the GQD/SiOx/C composite according to the silicon oxide content.

**Figure 6:**
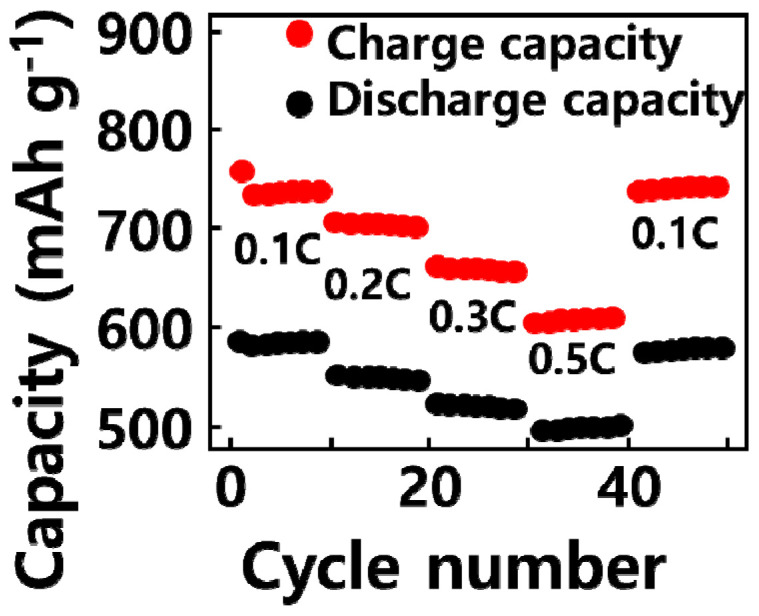
Rate capability at current rates from 0.1 C to 0.5 C, respectively.

Figure 6 provides information on the rate characteristics after changing the current speed (0.1 C, 0.2 C, 0.5 C, 1 C, and 2 C) of the secondary battery according to the silicon oxide content of the GQD/SiOx/C composite. As the content of silicon oxide increased from 0 to 15 wt%, the rate characteristic increased from 76 to 81. It can be seen that the rate characteristic improved as silicon oxide was added. Similar to the electrical conductivity analysis results in Figure 3, it can be seen that as silicon oxide was added to the composite, the electrical conductivity of the composite increased, and the rate characteristics improved. It is believed that the high electrical conductivity and high specific surface area of graphene quantum dots and carbon nanoparticles provided a path for lithium ions to move to the silicon oxide, which has a low electrical conductivity, thereby improving the rate characteristics [28].

**Figure 7:**
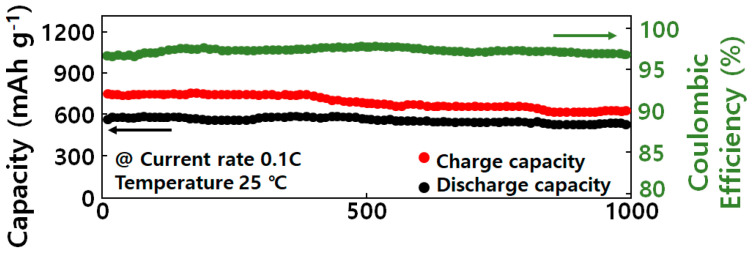
Electrochemical performance of the lithium-ion battery with a GQD/SiOx/C composite anode. Charge–discharge curve of the initial 1st cycles at a current rate of 0.1 C. Long-term cycling measurement at a current rate of 0.2 C.

Figure 7 shows the coulombic efficiency and cycle characteristics according to the silicon oxide content of the GQD/SiOx/C composite. To apply silicon-based anode materials, high coulombic efficiency is important. Generally, graphite is mixed with carbon-based materials because it has a high coulombic efficiency of 91 to 95%. The GQD/SiOx/C−0 composite had a low first Coulombic efficiency due to its irreversible capacity but showed a high Coulombic efficiency of over 94% in the second cycle because volume expansion of silicon oxide occurred due to the SEI layer on the electrode surface and the insertion and detachment of lithium ions. However, the internal cavity of the composite formed by graphene quantum dots and carbon nanoparticles buffered the volume change of silicon oxide and stabilized the structure. This is believed to be due to the cycle performance. As the silicon oxide content of the GQD/SiOx/C composite increased from 0 to 15 wt%, the charging capacity improved from 594 mAh/g to 867 mAh/g. The GQD/SiOx/C−0 composite shows higher capacity characteristics compared to existing graphite anode materials. This is believed to have improved the capacity by providing a storage path for lithium ions in the internal cavity through graphene quantum dots and carbon nanoparticles [29]. When the silicon oxide content was less than 10 wt%, the capacity-improving effect was minimal, but the initial efficiency increased compared to that of GQD/SiOx/C−0, probably due to the formation of an excessive SEI layer in the cavity formed by the high specific surface area of graphene quantum dots and carbon nanoparticles, resulting in an increase in irreversible capacity. So, when silicon oxide was added, the specific surface area decreased because the cavity volume decreased due to the sharing of empty space inside the composite. The reduced specific surface area reduced the irreversible capacity because the area that reacted with lithium ions became smaller. As the irreversible capacity decreased, the difference between initial charge and discharge capacity decreased, improving the initial efficiency. As a result, as the silicon oxide content of the GQD/SiOx/C composite increased from 0 to 15 wt%, the discharge capacity increased from 425 mAh/g to 595 mAh/g, and the initial efficiency increased from 75% to 83%. When the silicon-based composite was charged and discharged, the composite structure was broken due to the large volume change caused by the repeated insertion and detachment of lithium ions; an excessive SEI layer formed, reducing initial efficiency and cycle stability. To solve this problem, graphene quantum dots and carbon nanoparticles with a large specific surface area, which provide cavities inside the composite, were introduced, and the volume change of silicon oxide was alleviated to maintain the structure, resulting in stable cycle stability. However, when the content of silicon oxide was low compared to the contents of graphene quantum dots and carbon nanoparticles, the initial efficiency was low because of the cavity formed by the high specific surface area of the graphene quantum dots and carbon nanoparticles [30,31]. Therefore, it was confirmed that the initial efficiency can be improved when the silicon oxide content of the GQD/SiOx/C composite is more than 10 wt% greater than the contents of the graphene quantum dots and carbon nanoparticles. In addition, silicon forms an SEI layer through an alloying reaction with lithium ions, and irreversible capacitance occurs, reducing the initial efficiency.

## 3. Materials and Methods

### 3.1. Material Preparation

Binder pitch was synthesized using pylorsis fuel oil as a precursor in a 2 L reactor at 420 °C, 80 min, pressure 1 bar, and softening point of 130 °C. To manufacture the composite, graphene quantum dots and carbon nanoparticles were synthesized, and magnesium-added silicon oxide was used as a filler for the molded body. The mixing ratio of graphene quantum dots, carbon nanoparticles, silicon oxide, and binder pitch is shown in Table 1. A Thinky mixer, Thinky, Laguna Hills, CA, USA (2500 rpm, 90 min) was used to uniformly mix and disperse the materials, and a heated press (190 °C, 20 min) was used to improve the density of the mixed material to produce a molded body. The manufactured molded body was placed in the center of the furnace and carbonized by maintaining it at 600 °C for 5 h at a rate of 5 °C/min in an argon atmosphere. The carbonized molded body was pulverized and sonicated in tetrahydrofuran (THF) solvent for 20 min. Then, the dispersion solution was stirred using a thermal stirrer (300 rpm, 8 h) and dried at 80 °C to prepare a composite. The prepared composite was carbonized at 600 °C for 6 h in an argon atmosphere at a rate of 5 °C/min. The prepared composite was ground and impurities were removed to prepare a particle size of 10 μm or less, and the prepared composite was classified as GQD/SiOx/C according to the content of silicon oxide.

### 3.2. Characterization of Prepared Composite

To analyze the crystal structure of the manufactured composite and raw materials, an X-ray diffraction (XRD, Rigaku Max 2400 Co., Japan, Cu Kα-radiation λ = 1.54178) analysis was performed. The measurement range was 2θ = 20~80°, the step was 0.02°, and the step time was 2 s. The acceleration voltage was 40 kV, and the current was 100 mA. Through the XRD analysis results, the crystallinity of the composite raw materials and the coating layer of the composite were confirmed. Scanning electron microscope (SEM, Verios G4UC, Thermo Fisher Scientific, U.S.) analysis was performed to observe the degree of dispersion of silicon oxide inside the manufactured composite. After etching the composite through focused ion beam (FIB) milling, the inside of the composite was observed. The degree of dispersion and the content of silicon oxide according to the content of silicon oxide were confirmed. In addition, an energy-dispersive X-ray spectrometer (EDS, EM-09100IS, JEOL, Japan) analysis was performed to observe the type and content of elements in the FIB (NEOARM, JEOL, Japan)-milled composite. The constituent elements of the composite were confirmed through the analyzed elements, and the degree of dispersion was confirmed according to the content of silicon oxide. To analyze the electrochemical properties of the manufactured composite, a powder resistivity measurement system was used to analyze the electrical conductivity of the composite. The pressure was set in a range from 300 to 2000 kgf/cm^2^. The electrical conductivity of the silicon composite was measured, and the characteristics of electrical conductivity were analyzed by comparing the composite with composites under other conditions.

### 3.3. Lithium Secondary Battery Assembly and Electrochemical Characterization

An electrode was prepared to analyze the electrochemical properties of the GQD/SiOx/C composite according to the silicon oxide content. The electrode was mixed with the active material and water-based binder (CMC, SBR) at a weight ratio of 8.5:1.5 in a Thinky mixer (2500 rpm 2 min). The mixed slurry was coated onto copper foil to prepare a current-collector electrode; the electrode was dried in a vacuum oven at 120 °C for 8 h. The electrode density of the coated electrode was improved through a rolling process using a roll press; a half cell was manufactured using lithium metal as the electrode in a glove box. The cycle, rate characteristics, and impedance analysis of the fabricated half cell were evaluated using a WBCS 3000 battery cycler (Won A Tech, Inc., Daejeon, Republic of Korea).

## 4. Conclusions

In this study, silicon oxide was added to improve the capacity of lithium-ion battery anode material, and a composite was manufactured by adding graphene quantum dots and carbon nanoparticles, which are structural stabilizers, to alleviate the volume change of the added silicon oxide. The prepared composite was subjected to physical property analysis, FIB-SEM, EDS, XRD, powder resistance, and electrochemical characterization, i.e., cycle characteristics, rate velocity, and impedance analysis. As a result of the physical property analysis, the crystallinity and elements of the anode material were confirmed as depending on the content of silicon oxide. Additionally, as a result of electrochemical property evaluation, the initial capacity of the GQD/SiOx/C composite was found to be 768 mAh/g and the initial efficiency was 83%. At a high current rate of 2 C, the rate characteristic was 81 compared to an initial value of 0.1 C. When graphene quantum dots and carbon nanoparticles, which are structural stabilizers, were added to the anode material of GQD/SiOx/C, the electric capacity, initial efficiency, and rate characteristics were improved by the addition of silicon oxide. The addition of graphene quantum dots and carbon nanoparticles formed a cavity inside the silicon anode material and buffered the volume expansion of the silicon oxide, leading to structural stabilization of the anode material. The increase in the electrical conductivity of the silicon composite via the addition of graphene quantum dots and carbon nanoparticles improved the rate characteristics. In this study, a silicon oxide content of 15 wt% compared to the existing graphite anode material was judged to be the optimal condition, and the applicability of the appropriately mixed GQD/SiOx/C composite as a high-performance secondary battery anode material was confirmed.

## Figures and Tables

**Table 1:** GQD/SiOx/C composite synthesis conditions of materials.

	SiOx (Weight %)	SiOx (g)	Graphene Quantum Dot (g)	Carbon Nanoparticle (g)
GQD/SiOx/C-0	0	0	2.3	1.8
GQD/SiOx/C-5	5	0.54	2.3	1.8
GQD/SiOx/C-10	10	1.1	2.3	1.8
GQD/SiOx/C-15	15	1.52	2.3	1.8
GQD/SiOx/C-30	30	3.04	2.3	1.8

**Table 2:** Specific capacity of GQD/SiOx/C composite anode.

	1st Charge Capacity (mAh/g)	1st Discharge Capacity (mAh/g)	Initial Efficient (%)
GQD/SiOx/C-0	594	425	71.6
GQD/SiOx/C-5	615	445	72.4
GQD/SiOx/C-10	634	493	77.9
GQD/SiOx/C-15	768	595	77.5
GQD/SiOx/C-30	867	591	68.2

## Data Availability

The data presented in this study are available in article and Supplementary Materials.

## Supplementary Materials

The following supporting information can be downloaded at: https://www.mdpi.com/article/10.3390/molecules29112578/s1, Figure S1: Electrochemical performance of the GQD/SiOx/C -15 composite: Differential capacity plots; Figure S2: Electrochemical performance of the GQD/SiOx/C -15 composite: Discharge-charge profiles at different current densities. Figure S3: Raman spectra of GQD/SiOx/C composite. Figure S4: SEM and TEM image of GQD/SiOx/C composite. Figure S5: TEM image of GQD/SiOx/C composite. Figure S6: TGA curves of GQD/SiOx/C composite at different weight ratios of SiOx/C to GQD. Table S1: Electrochemical performances of various SiOx-based anodes for LIBs. Refs. [32,33,34,35,36,37,38] are cited in the Supplementary Materials.

## References

[1] Chang W., Park C., Kim J., Kim Y., Jeong G., Sohn H. (2012). Quartz (SiO_2_): A new energy storage anode material for Li-ion batteries. Energy Environ. Sci..

[2] Choi G., Kim J., Kang B. (2019). Understanding limited reversible capacity of a SiO electrode during the first cycle and its effect on initial coulombic efficiency. Chem. Mater..

[3] Cui J., Cui Y., Li S., Sun H., Wen Z., Sun J. (2016). Microsized porous SiO_x_@C composites synthesized through aluminothermic reduction from rice husks and used as anode for lithium-ion batteries. ACS Appl. Mater. Interfaces.

[4] Fu J., Liu H., Liao L., Fan P., Wang Z., Wu Y. (2018). Ultrathin Si/CNTs paper-like composite for flexible li-ion battery anode with high volumetric capacity. Front. Chem..

[5] Guo C., Wang D., Liu T., Zhu J., Lang X. (2014). A three-dimensional SiO_x_/C@RGO nanocomposite as a high energy anode material for lithium-ion batteries. J. Mater. Chem. A.

[6] Hang T., Mukoyama D., Nara H., Yokoshima T., Momma T., Li M. (2014). Electrochemical impedance analysis of electrodeposited Si–O–C composite thick film on Cu microcones-arrayed current collector for lithium ion battery anode. J. Power Sources.

[7] Hou X., Zhang M., Wang J., Hu S., Liu X., Shao Z. (2015). High yield and low-cost ball milling synthesis of nano-flake Si@SiO_2_ with small crystalline grains and abundant grain boundaries as a superior anode for Li-ion batteries. J. Alloys Compd..

[8] Huang X., Pu H., Chang J., Cui S., Hallac P.B., Jiang J. (2013). Improved cyclic performance of Si anodes for lithium-ion batteries by forming intermetallic interphases between Si nanoparticles and metal microparticles. ACS Appl. Mater. Inter..

[9] Hubaud A.A., Yang Z., Schroeder D.J., Dogan F., Trahey L., Vaughey J.T. (2015). Interfacial study of the role of SiO_2_ on Si anodes using electrochemical quartz crystal microbalance. J. Power Sources.

[10] Hwa Y., Park C., Sohn H. (2013). Modified SiO as a high-performance anode for Li-ion batteries. J. Power Sources.

[11] Jia H., Zheng J., Song J., Luo L., Yi R., Estevez L. (2018). A novel approach to synthesize micrometer-sized porous silicon as a high-performance anode for lithium-ion batteries. Nano Energy.

[12] Kataoka R., Oda Y., Inoue R., Kitta M., Kiyobayashi T. (2016). Highstrength clad current collector for silicon-based negative electrode in lithium ion battery. J. Power Sources.

[13] Kim J., Park C., Kim H., Kim Y., Sohn H. (2011). Electrochemical behavior of SiO anode for Li secondary batteries. J. Electrochem. Chem..

[14] Kim M.K., Jang B.Y., Lee J.S., Kim J.S., Nahm S. (2013). Microstructures and electrochemical performances of nano-sized SiO_x_ (1.18 ≤ x ≤ 1.83) as an anode material for a lithium (Li)-ion battery. J. Power Sources.

[15] Krywko-Cendrowska A., Strawski M., Szklarczyk M. (2013). Low temperature electrodeposition of SiO_x_ films photoactive in water solution. Electrochim. Acta.

[16] Lee M., Yoon D., Lee U.J., Umirov N., Mukanova A., Bakenov Z. (2019). The electrochemical performances of n-type extended lattice spaced Si negative electrodes for lithium-ion batteries. Front. Chem..

[17] Li M., Li J., Li K., Zhao Y., Zhang Y., Gosselink D. (2013). SiO_2_/Cu/polyacrylonitrile-C composite as anode material in lithium ion batteries. J. Power Sources.

[18] Li M., Zeng Y., Ren Y., Zeng C., Gu J., Feng X. (2015). Fabrication and lithium storage performance of sugar apple-shaped SiO_x_@C nanocomposite spheres. J. Power Sources.

[19] Liang B., Liu Y., Xu Y. (2014). Silicon-based materials as high capacity anodes for next generation lithium ion batteries. J. Power Sources.

[20] Lv P., Zhao H., Gao C., Du Z., Wang J., Liu X. (2015). SiO_x_C dual-phase glass for lithium ion battery anode with high capacityand stable cycling performance. J. Power Sources.

[21] Lv P., Zhao H., Wang J., Liu X., Zhang T., Xia Q. (2013). Facile preparation and electrochemical properties of amorphous SiO_2_/C composite as anode material for lithium ion batteries. J. Power Sources.

[22] Rahaman O., Mortazavi B., Rabczuk T. (2016). A first-principles study on the effect of oxygen content on the structural and electronic properties of silicon suboxide as anode material for lithium ion batteries. J. Power Sources.

[23] Rahman M.A., Song G., Bhatt A.I., Wong Y.C., Wen C. (2016). Nanostructured silicon anodes for high-performance lithium-ion batteries. Adv. Funct. Mater..

[24] Si Q., Hanai K., Ichikawa T., Phillipps M.B., Hirano A., Imanishi N. (2011). Improvement of cyclic behavior of a ball-milled SiO and carbon nanofiber composite anode for lithium-ion batteries. J. Power Sources.

[25] Song K., Yoo S., Kang K., Heo H., Kang Y., Jo M. (2013). Hierarchical SiO_x_ nano for Li-ion battery anodes with structural stability and kinetic enhancement. J. Power Sources.

[26] Su X., Wu Q., Li J., Xiao X., Lott A., Lu W. (2014). Siliconbased nanomaterials for lithium-ion batteries: A review. Adv. Energy Mater..

[27] Tao H., Fan L., Qu X. (2012). Facile synthesis of ordered porous Si@C nanorods as anode materials for Li-ion batteries. Electrochim. Acta.

[28] Vengudusamy B., Grafl A., Preinfalk K. (2014). Influence of silicon on the wear properties of amorphous carbon under dry and lubricated conditions. Tribol. Lett..

[29] Wang D., Gao M., Pan H., Wang J., Liu Y. (2014). High performance amorphous-Si@SiO_x_/C composite anode materials for Li-ion batteries derived from ball-milling and in situ carbonization. J. Power Sources.

[30] Wang J., Wang C., Zhu Y., Wu N., Tian W. (2015). Electrochemical stability of optimized Si/C composites anode for lithium-ion batteries. Ionics.

[31] Wang J., Zhao H., He J., Wang C., Wang J. (2011). Nano-sized SiO_x_/C composite anode for lithium ion batteries. J. Power Sources.

[32] Zhou X., Tang J., Yang J., Xie J., Ma L. (2013). Silicon@carbon hollowcore–shell heterostructures novel anode materials for lithium ion batteries. Electrochim. Acta.

[33] Xie J., Wang G., Huo Y., Zhang S., Cao G., Zhao X. (2014). Nanostructured silicon spheres prepared by a controllable magnesiothermicreduction as anode for lithium ion batteries. Electrochim. Acta.

[34] Xia M., Li Y., Wu Y., Zhang H., Yang J., Zhou N., Zhou Z., Xiong X. (2019). Improvingthe electrochemical properties of a SiO@C/graphite composite anode for high_energy lithium-ion batteries by adding lithium fluoride. Appl. Surf. Sci..

[35] Xiao L., Wu D., Han S., Huang Y., Li S., He M., Zhang F., Feng X. (2013). Self-assembledFe_2_O_3_/graphene aerogel with high lithium storage performance. ACS Appl. Mater. Interfaces.

[36] Doh C.H., Park C.W., Shin H.M., Kim D.H., Chung Y.D., Jin B.S., Kim H.S., Veluchamy A. (2008). A new SiO/C anode composition for lithium-ion battery. J. Power Sources.

[37] Wu W., Shi J., Liang Y., Liu F., Peng Y., Yang H. (2015). A low-cost and advanced SiOx–C composite with hierarchical structure as an anode material for lithium-ion batteries. Phys. Chem. Chem. Phys..

[38] Kim S.O., Manthiram A. (2015). A facile, low-cost synthesis of high-performance silicon-based composite anodes with high tap density for lithium-ion batteries. J. Mater. Chem. A.

