# Supplementary material for: SiOx/C Composite Anode for Lithium-Ion Battery with Improved Performance Using Graphene Quantum Dots and Carbon Nanoparticles
Source: Molecules. 2024 May 30;29(11):2578. doi: 10.3390/molecules29112578 (PMC11174013; doi:10.3390/molecules29112578)
Supplement: Supplementary file 1 [file molecules-29-02578-s001.zip › molecules-2966829-supplementary.pdf]

Supplementary Information for

SiOx/C composite anode for lithium-ion battery with improved performance using graphene quantum dots and carbon nano-particles

Sung Won Hwang 1,*

[1]Department of System Semiconductor Engineering, Sangmyung University, Cheonan 31066, South Korea

*Corresponding authors: sungwon@smu.ac.kr

Supplementary Figures

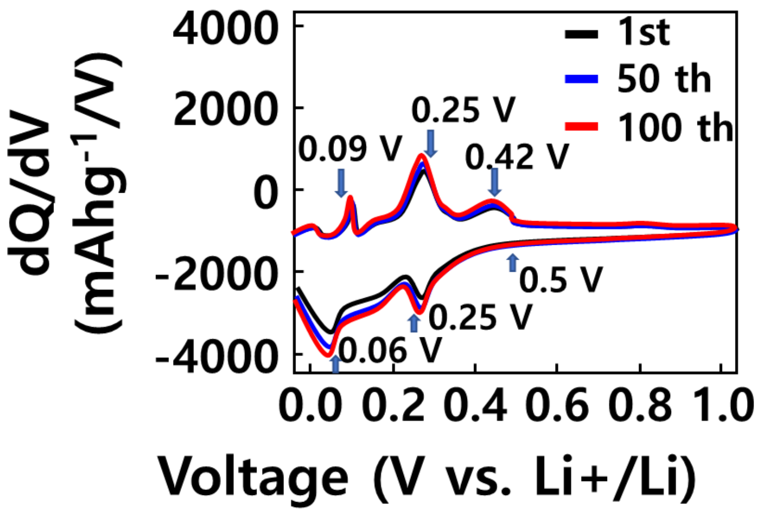

Figure S1. Electrochemical performance of the GQD/SiOx/C -15 composite: Differential capacity plots.

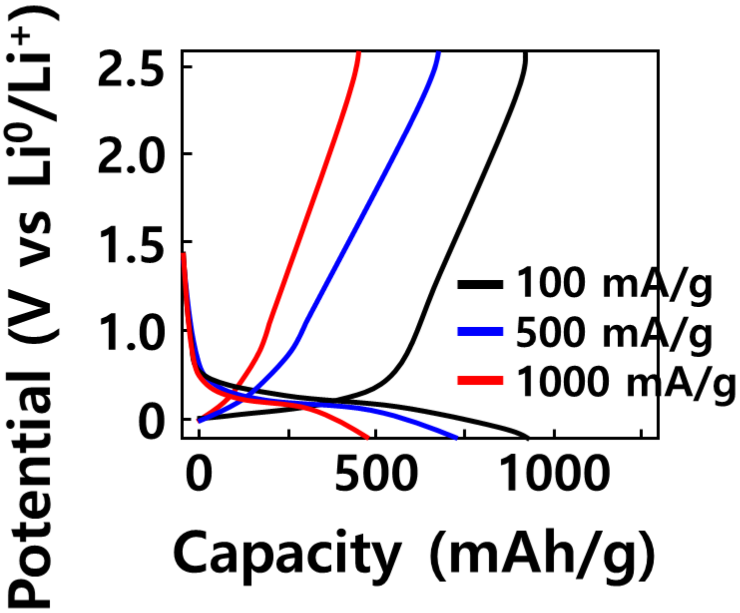

Figure S2. Electrochemical performance of the GQD/SiOx/C -15 composite: Discharge-charge profiles at different current densities.

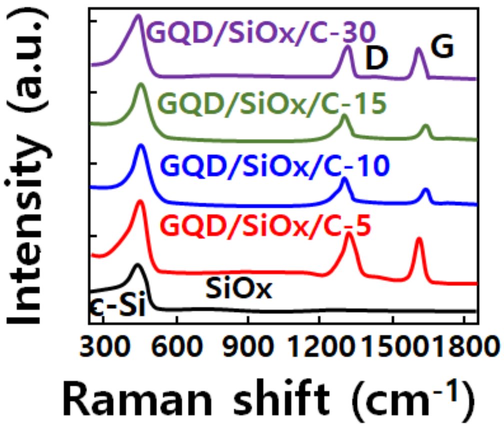

Figure S3. Raman spectra of GQD/SiOx/C composite.

Figure S3 compares the Raman spectra of SiOx and the as-prepared composites. The composite showed the lowest defect-related D-band at 1,348 cm−1. For the pristine SiOx, the bands ranging from 400 to 500 cm−1 are assigned to amorphous SiO (Guo et al., 2014; Rahaman et al., 2016. The Si peak appears in the Raman spectra of GQD/SiOx/C composites. This is consistent with the XRD results as shown in Figure 1. Compared with pure SiO, there is no distinct change of amorphous SiO peak in the Raman spectra of three composites. Concerning the typical D-band at 1,348 cm−1 and G-band at 1,582 cm−1 in the Raman spectrum of GQD/SiOx/C composite, which confirms the transformation of asphalt into disordered carbon. Besides, the Raman spectra of GQD/SiOx/C composites show similar D-band and G-band peaks with that of composite, despite the addition of graphite (Xiao et al., 2013; Vengudusamy et al., 2014).

Carbon nanoparticles and silicon oxide particles are uniformly distributed in the composite.

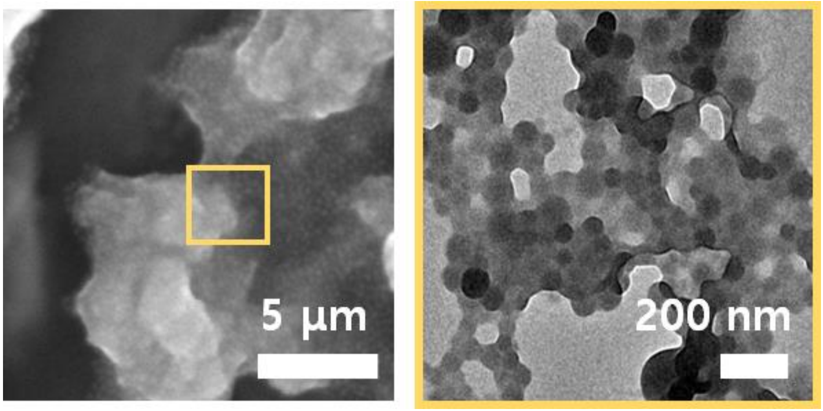

Figure S4. SEM and TEM image of GQD/SiOx/C composite.

The GQD/SiOx/C composite is further characterized by SEM and TEM.

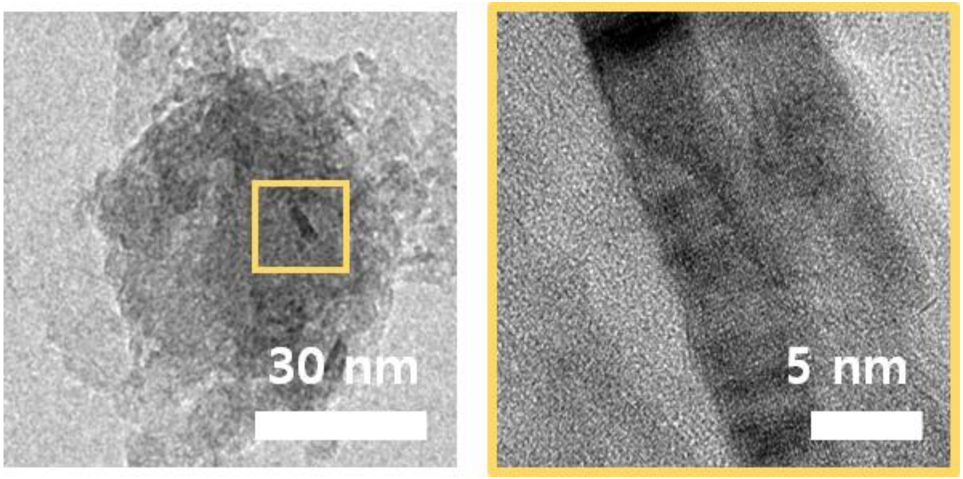

Figure S5. TEM image of GQD/SiOx/C composite.

Primary particles of 30 to 50 nm were aggregated. The primary particles of GQD/SiOx/C-15 composite is further characterized by TEM. Figure S5 shows the SiOx is evenly distributed over the graphite sheets. As seen in Figure 4, the SiOx is wrapped with amorphous carbon layer of the thickness of ~5 nm. Overall, the multi-phase microstructure containing amorphous SiOx plates and different carbon structures is formed in the GQD/SiOx/C composites.

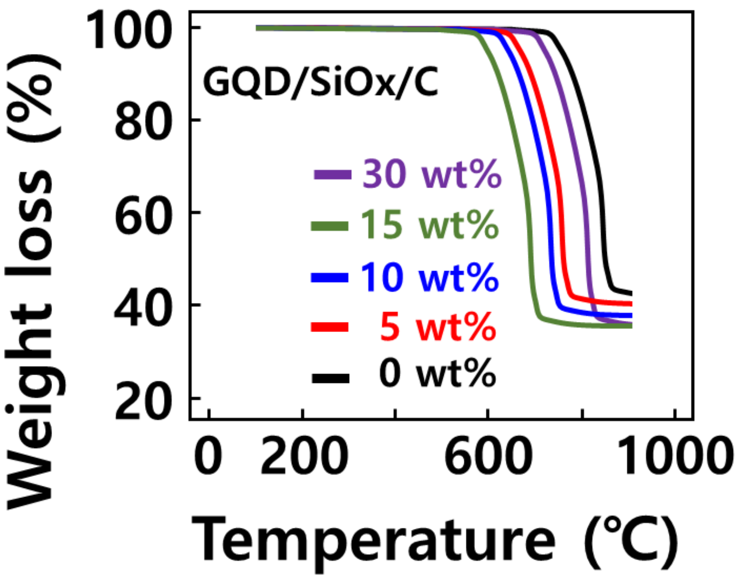

Figure S6. TGA curves of GQD/SiOx/C composite at different weight ratios of SiOx/C to GQD.

Structures	Charge capacity (mAhg-1)	Current density (mA g-1)	Cycle number	Coulombic efficiency first time (%)	Ref.
**GQD/SiOx/C nanocomposite**	768	100	1000	77.5	This work
**SiO/C**	688	100	30	44.5	11
**SiO/CNFs**	700	100	200	35.7	12
**SiOx-C**	674.8	100	100	77	13
**SiOx/C**	720	100	350	69	14

Table S1. Electrochemical performances of various SiOx-based anodes for LIBs.

Reference

1. Zhou, X., Tang, J., Yang, J., Xie, J., and Ma, L. (2013). Silicon@carbon hollow core–shell heterostructures novel anode materials for lithium ion batteries. Electrochim. Acta 87, 663–668

2. Hubaud, A. A., Yang, Z., Schroeder, D. J., Dogan, F., Trahey, L., and Vaughey, J. T. (2015). Interfacial study of the role of SiO2 on Si anodes using electrochemical quartz crystal microbalance. J. Power Sources 282, 639–644.

3. Tao, H., Fan, L., and Qu, X. (2012). Facile synthesis of ordered porous Si@C nanorods as anode materials for Li-ion batteries. Electrochim. Acta 71, 194–200.

4. Xie, J., Wang, G., Huo, Y., Zhang, S., Cao, G., and Zhao, X. (2014). Nanostructured silicon spheres prepared by a controllable magnesiothermic reduction as anode for lithium ion batteries. Electrochim. Acta 135, 94–100.

5. Hang, T., Mukoyama, D., Nara, H., Yokoshima, T., Momma, T., Li, M., et al. (2014). Electrochemical impedance analysis of electrodeposited Si–O–C composite thick film on Cu microcones-arrayed current collector for lithium ion battery anode. J. Power Sources 256, 226–232.

6. Cui, J., Cui, Y., Li, S., Sun, H., Wen, Z., and Sun, J. (2016). Microsized porous SiOx@C composites synthesized through aluminothermic reduction from rice husks and used as anode for lithium-ion batteries. ACS Appl. Mater. Inter. 8, 30239–30247.

7. Xia, M., Li, Y., Wu, Y., Zhang, H., Yang, J., Zhou, N., et al. (2019). Improving the electrochemical properties of a SiO@C/graphite composite anode for high energy lithium-ion batteries by adding lithium fluoride. Appl. Surf. Sci. 480, 410–418.

8. Guo, C., Wang, D., Liu, T., Zhu, J., and Lang, X. (2014). A three dimensional SiOx/C@RGO nanocomposite as a high energy anode material for lithium-ion batteries. J. Mater. Chem. A 2, 3521–3527.

9. Rahaman, O., Mortazavi, B., and Rabczuk, T. (2016). A first-principles study on the effect of oxygen content on the structural and electronic properties of silicon suboxide as anode material for lithium ion batteries. J. Power Sources 307, 657–664.

10. Xiao, L., Wu, D., Han, S., Huang, Y., Li, S., He, M., et al. (2013). Self-assembled Fe2O3/graphene aerogel with high lithium storage performance. ACS Appl. Mater. Interfaces 5, 3764–3769.

11. C. H. Doh, C. W. Park, H. M. Shin, D. H. Kim, Y. D. Chung, S. I. Moon, B. S. Jin, H. S. Kim and A. Veluchamy, A new SiO/C anode composition for lithium-ion battery J. Power Sources, 2008, 179, 367–370.

12. Q. Si, K. Hanai, T. Ichikawa, M. B. Phillipps, A. Hirano, N. Imanishi, O. Yamamoto and Y. Takeda, Improvement of cyclic behavior of a ball-milled SiO and carbon nanofiber composite anode for lithium-ion batteries J. Power Sources, 2011, 196, 9774–9779.

13. W. Wu, J. Shi, Y. Liang, F. Liu, Y. Peng and H. Yang, A low-cost and advanced SiOx–C composite with hierarchical structure as an anode material for lithium-ion batteries Phys. Chem. Chem. Phys., 2015, 17, 13451–13456.

14. S. O. Kim and A. Manthiram, A facile, low-cost synthesis of high-performance silicon-based composite anodes with high tap density for lithium-ion batteries J. Mater. Chem. A, 2015, 3, 2399–2406.
